# Bennett's Fracture

**Published:** 1904-04-09

**Authors:** 


### BENNETT'S FRACTURE.

In 1881 attention was first directed by Professor Bennett, of Dublin, to the fact that fracture through the base of the metacarpal bone of the thumb is a common injury. He held that it is the commonest fracture of the metacarpus; that it may be readily recognised clinically, but is constantly overlooked, greatly to the disadvantage of the patients. Since that time but few instances of the injury have been recorded, and but little attention appears to have been paid to the subject. Alexander Miles and J. W. Struthers[1] now come forward to support Bennett's views concerning the frequency and nature of the fracture. Since 1900 they have seen 15 clinical cases of fracture of the base of the first metacarpal bone, and have been able to find two specimens of undoubted old-standing cases of the injury and two specimens showing changes probably due to Bennett's fracture followed by osteoarthritis of the neighbouring joint. They describe the fracture as a chipping off of the palmar projection of the base of the metacarpal bone of the thumb, with a varying amount of upward and backward displacement of the rest of the bone, the short palmar fragment retaining its normal relation to the trapezium. The injury is caused by force applied to the tip of the outstretched thumb, or to the end of the first phalanx when the terminal phalanx is flexed, and may be produced by striking the thumb in falling or in delivering a blow with the closed fist. The line of fracture will vary according as the metacarpal bone is flexed or extended on the trapezium at the moment of the accident. In full flexion the summit of the convexity of the trapezium rests more towards the dorsal margin of the articular surface of the metacarpal bone than in full extension, when it rests more towards the palmar margin. The line of fracture, therefore, is nearer the dorsal aspect of the bone when the accident happens with the metacarpal bone flexed, and nearer the palmar surface when the accident happens with the metacarpal extended. In many of the cases which were observed by Miles and Struthers the patients at first regarded the injury as a trivial one, and did not apply for treatment till the continued pain and weakness in the injured thumb made it clear to them that they were not merely suffering from a sprain. The authors note that crepitus may still be observed in patients who first come for treatment some weeks after the original injury. Only once were they unable to detect crepitus within four weeks after the fracture had occurred.

They state that there is no difficulty in distinguishing fracture of the metacarpal bone from dislocation backwards. The facts that the deformity can be reduced, that the reduction is accompanied by crepitus, and that the displacement returns when the reducing force is removed, all serve to distinguish the fracture from a dislocation. In recent cases the displacement can easily be corrected by making traction on the thumb in the abducted and fully extended position, and at the same time making pressure over the dorsal aspect of the metacarpal bone. The main difficulty consists in maintaining the fragments in apposition. The best splint is a palmar one extending from the tip of the thumb across the base of the hand, and projecting beyond the ulnar side of the wrist. The lower end of the splint is first fixed firmly to the end of the thumb, which is then fully abducted and extended, the fragments being suitably manipulated. The abduction and extension are kept up by applying a bandage round the wrist and hand so as to catch in the end of the splint and push it downwards towards the radial side. In cases where the displacement cannot be reduced, nothing is to be gained by fixing the thumb, and the patient should be encouraged from the first to persevere with passive and active movement of the thumb at the metacarpal joint. The prognosis is almost uniformly good as regards function though a small amount of visible deformity may remain.

[1] Edin. Med. Jour. April, 1904.

